# CD82 inhibits canonical Wnt signalling by controlling the cellular distribution of β-catenin in carcinoma cells

**DOI:** 10.3892/ijo.2012.1671

**Published:** 2012-10-17

**Authors:** SATOMI CHIGITA, TSUYOSHI SUGIURA, MASAKAZU ABE, YOSUKE KOBAYASHI, MIYUKI SHIMODA, MEGUMI ONODA, KANEMITSU SHIRASUNA

**Affiliations:** Division of Maxillofacial Diagnostic and Surgical Sciences, Department of Oral and Maxillofacial Surgery, Graduate School of Dental Science, Kyushu University, Higashi-ku, Fukuoka 812-8582, Japan

**Keywords:** CD82, β-catenin, Wnt signalling, cancer cell adhesion

## Abstract

We have recently unravelled a novel function for CD82 in E-cadherin-mediated cellular adhesion. CD82 inhibits β-catenin tyrosine phosphorylation and stabilizes E-cadherin-β-catenin complexes at the cell membrane. This function inhibits cancer cell dissociation from the primary cancer nest and limits metastasis. In this study, we focused on the effect of CD82 on the Wnt/β-catenin (canonical) pathway, which controls the cellular distribution of β-catenin. CD82 had no effect on the expression of Wnt proteins but led to significant downregulation of Frizzled (Fzd) 2, 3, 5, 7 and 9, suggesting downregulation of the Wnt/β-catenin pathway. CD82 also inhibited phosphorylation of β-catenin at Ser45, Ser33, Ser37 and Thr41 by downregulation of glycogen synthase kinase-3β (GSK-3β) and kinase casein kinase 1α (CK1α). Downregulation of GSK-3β and CK1α also led to accumulation of β-catenin in the cytoplasm or at the cell membrane. CD82 translocated β-catenin to the cell membrane, suggesting that CD82 strengthens the interaction between E-cadherin and β-catenin. We concluded that CD82 attenuates Wnt signalling by controlling β-catenin cellular distribution at multiple levels: i) inhibition of β-catenin nuclear translocation by downregulation of Fzd receptor proteins; ii) accumulation of β-catenin at the cell membrane by downregulation of GSK-3β and CK1α; and iii) stabilization of the E-cadherin-β-catenin complex.

## Introduction

Metastasis is a multi-step cascade involving the migration of tumour cells from their site of origin, evasion from host defence systems, subsequent seeding at distant organs and growth of secondary tumours. The first step of metastasis is migration of the tumour cells from the primary tumour nest. In this process, tumour cells are required to loosen their homophilic cell adhesion, enabling tumour cells to escape from the tumour nest. Classic cadherins interact homophilically with cadherins of neighbouring cells to form adherens junctions, which serve both as mechanical linkages between cells and signalling hubs that relay information from the extracellular environment (1–5). Epithelial cadherin, or E-cadherin, is thought to be a tumour-suppressor molecule largely because it is frequently downregulated in carcinomas (6–8). Loss of E-cadherin has also been shown to be a hallmark of epithelial-mesenchymal transition (EMT) in cancer cells and to directly correlate with malignant phenotype and poor prognosis (9–11).

The cellular distribution of β-catenin has a major influence on the control of the malignant phenotype of cancer cells. Accumulation of β-catenin in the nucleus correlates with poor prognosis in many types of cancer. Upon translocation into the nucleus, β-catenin forms complexes with members of the T cell factor (TCF)/lymphoid enhancer factor (LEF) family of transcription factors (12–14), leading to the activation of responsive genes involved in cell proliferation, differentiation and other malignant phenotypes (15,16). Cytosolic β-catenin is the principal mediator of canonical Wnt signalling (17,18). In the absence of an extracellular Wnt ligand, cytosolic β-catenin is phosphorylated at Ser45 by the priming kinase casein kinase 1α (CK1α) and incorporated into a cytosolic protein complex containing Axin, the adenomatous polyposis coli gene product (APC) and glycogen synthase kinase-3β (GSK-3β) (19). Axin and APC serve as scaffolding proteins that enable GSK-3β to phosphorylate β-catenin at residues Ser33, Ser37 and Thr41 (20), thereby targeting it for ubiquitination by β-TrCP (β-transducin repeat-containing homologue protein) and subsequent degradation in the proteasome. Cytosolic β-catenin protein levels are thus kept low in the absence of Wnt ligand stimulation. Binding of a Wnt ligand to its co-receptors Frizzled (Fzd) and low-density lipoprotein (LDL) receptor-related protein (LRP) 5/6 results in the activation of the Dishevelled (Dvl) protein, which then inhibits GSK-3β-mediated phosphorylation of β-catenin. Cytosolic β-catenin is thus stabilized and is able to accumulate. This pool of β-catenin translocates to the nucleus and promotes malignancy by binding to TCF/LEF (18,19). In addition to its function in Wnt signalling, β-catenin is a component of the cadherin-based adherens junction complexes formed at cell-cell adhesion sites. β-catenin binds the cytoplasmic domain of cadherin and acts as a structural protein by linking cell-surface cadherins to the actin cytoskeleton (21). By sequestering β-catenin at the membrane, cadherins modulate the signalling properties of cytosolic β-catenin, creating a finely tuned balance between Wnt signalling and cell-cell adhesion (22–24).

Tetraspanins, or TM4SF (transmembrane 4 superfamily) proteins, compose a large group of cell-surface transmembrane proteins, some of which can form complexes with integrins. Several tetraspanins appear to be particularly relevant to tumour cell metastasis (25,26). CD82 (CD82/KAI-1), a member of the tetraspanin superfamily, was originally identified as an accessory molecule in T cell activation (27). The role played by CD82 in cancer progression was discovered during a genetic screen to identify metastasis-suppressor genes (28). Ample evidence suggests that CD82 acts as a broad-spectrum suppressor of invasion and metastasis during the progression of various solid tumours. In malignant solid tumours, the detection of CD82 expression indicates a better prognosis for cancer patients, whereas the downregulation or loss of CD82 expression is commonly associated with clinically advanced cancers (29,30). Our previous studies indicate that CD82 negatively controls cancer cell migration and proteinase secretion by regulating cell signalling events, particularly those mediated by receptor tyrosine kinase (RTK) and phosphoinositide 3-kinase (PI3K) (25,31,32). These results suggest that the chief function of CD82 involves the normalization of uncontrolled malignant phenotypes in cancer cells by regulating the expression of cell-surface molecules. Recently, we have unravelled a novel function for CD82 in E-cadherin-mediated cellular adhesion (33). CD82 inhibits β-catenin tyrosine phosphorylation and stabilizes E-cadherin-β-catenin complexes at the cell membrane. CD82 favours homocellular adhesion and controls the cellular distribution of β-catenin. This function inhibits cancer cell dissociation from the primary cancer nest and limits metastasis (33).

In this study, we investigated the effect of CD82 on the canonical Wnt pathway (also important in the control of β-catenin cellular distribution) and showed that CD82 inhibits Wnt signalling in a multifunctional way.

## Materials and methods

### Antibodies

Mouse monoclonal antibodies against KAI-1 (G-2), E-cadherin (H-106) and rabbit polyclonal antibodies against KAI-1 (C-16) were purchased from Santa Cruz Biotechnology (Santa Cruz, CA, USA). Rabbit polyclonal and mouse monoclonal antibodies against β-catenin were purchased from Upstate Laboratories (Temecula, CA, USA). The rabbit monoclonal antibody against phospho-β-catenin (pSer33/pSer37) was purchased from Upstate Laboratories. Mouse monoclonal antibodies against phospho-β-catenin (pThr41 and pSer45) were purchased from Sigma-Aldrich (St. Louis, MO, USA). The sheep polyclonal antibody against CK1α was purchased from R&D Systems (Minneapolis, MN, USA) and the rabbit monoclonal antibody against GSK-3β, from Cell Signalling Technology (Danvers, MA, USA).

The anti-Wnt protein antibodies used in this study were as follows: anti-Wnt1 (rabbit polyclonal; GeneTex, Inc., Irvine, CA, USA), anti-Wnt2 (rabbit polyclonal; ProteinTech Group, Chicago, IL, USA), anti-Wnt2B (rabbit polyclonal; AVIVA Systems Biology, San Diego, CA, USA), anti-Wnt3 (mouse monoclonal; LifeSpan Biosciences, Seattle, WA, USA), anti-Wnt3a (rabbit polyclonal; Acris Antibodies, Herford, Germany), anti-Wnt4 (rat monoclonal; Acris Antibodies), anti-Wnt5a (rabbit polyclonal; LifeSpan Biosciences), anti-Wnt5b (rabbit polyclonal; Novus Biologicals, Littleton, CO, USA), anti-Wnt6 (rabbit polyclonal; Novus Biologicals), anti-Wnt7a and anti-Wnt7b (goat polyclonal; R&D Systems), anti-Wnt8a (rabbit polyclonal; Novus Biologicals), anti-Wnt8b (rat monoclonal; Acris Antibodies), anti-Wnt9a (goat polyclonal; R&D Systems), anti-Wnt9b (rabbit polyclonal; GeneWay Biotech, Inc., San Diego, CA, USA), anti-Wnt10a (rabbit polyclonal; Novus Biologicals) and anti-Wnt10b (Wnt12) (rabbit polyclonal; LifeSpan Biosciences).

The anti-Fzd protein antibodies used in this study were as follows: rabbit polyclonal antibodies against Fzd1, Fzd3, Fzd4, Fzd6, Fzd7, Fzd8, Fzd9 and Fzd10 (GenWay Biotech, Inc.) and rabbit polyclonal antibodies against Fzd2 and Fzd5 (GeneTex, Inc.).

### Cell cultures

The human cell line h1299 (non-small-cell lung carcinoma) and its transfectant derivatives (h1299/zeo and h1299/CD82) were established in our laboratory by transfection of a control vector or CD82 cDNA and cell sorting-based clone selection, as described previously (50). Further, h1299/zeo is a mock transfectant with weak CD82 expression and h1299/CD82 overexpresses CD82. The protein levels of CD82 in h1299/CD82 cells, as assayed by immunoblotting, are 20 times higher but its cell surface expression, as assessed by flow cytometry, is only ∼9-fold higher than that in the wild-type or h1299/zeo cells. The cell lines used in this study were maintained in Dulbecco’s modified Eagle’s medium (DMEM; Sigma) supplemented with 10% fetal bovine serum (FBS; ICN Biomedicals, Aurora, OH, USA) and 2 mM L-glutamine at 37°C and in an atmosphere of 5% CO_2_.

### Transfection of short hairpin RNA (shRNA)

The h1299/CD82-sh.control and h1299/CD82-sh.CD82 cell lines were generated by Lipofectamine (Invitrogen Life Technologies, Carlsbad, CA, USA) transfection of h1299/CD82 cells with pLKO.1-puro Control Vector (Sigma) and pLKO.1-puro/sh.CD82 (NM_002231; Sigma), respectively. Colonies that showed resistance to puromycin (Sigma) were pooled from the individual transfection experiments. The expression levels of CD82 in shRNA-transfected h1299 cells were monitored by reverse transcriptase-polymerase chain reaction (RT-PCR) and immunoblotting. The h1299/CD82-sh.control and h1299/CD82-sh.CD82 cells were maintained in DMEM containing 10% FBS and 2 *μ*g/ml puromycin.

### Immunoblot analysis

Cell lysates for immunoblotting were prepared in cell lysis buffer [1% Triton X-100, 2 mM sodium orthovanadate, 500 mM NaCl, 10 mM MgCl_2_, 10 *μ*g/ml leupeptin, 10 *μ*g/ml aprotinin, 1 mM PMSF, 50 mM Tris-HCl (pH 7.2)]. Subcellular fractionation was performed using the ProteoExtract^®^ Subcellular Proteome Extraction kit from Merck (Darmstadt, Germany) according to the manufacturer’s instructions.

The samples were resolved by sodium dodecyl sulphate-polyacrylamide gel electrophoresis (SDS-PAGE), transferred to a nitrocellulose membrane (Bio-Rad, Hercules, CA, USA) and incubated with specific primary antibodies. Protein bands were visualized using horseradish peroxidise (HRP)-conjugated secondary antibodies and Enhanced Chemiluminescence Reagent (Amersham Pharmacia Biotech, Piscataway, NJ, USA). The bands were scanned by computer-assisted densitometry (ChemiDoc XRS-J; Bio-Rad) and analysed using the Quantity One software (Bio-Rad).

### Real-time RT-PCR

Total RNA was extracted from h1299 cells by using TRIzol (Invitrogen, Carlsbad, CA, USA) and used for first-strand cDNA synthesis. The mRNA levels were measured in triplicate using a real-time PCR system with the Brilliant SYBR Green qPCR kit (Stratagene, La Jolla, CA, USA). Specific primers for GSK-3β and CK1α were as follows: GSK-3β (F: 5′-GG TCTATCTTAATCTGGTGCTGG-3′ and R: 5′-AGGTTCTGC GGTTTAATATCCC-3′) and CK1α (F: 5′-F:GGAAAAGAAGC ATGACTGTTAG-3′ and R: 5′-TCTGTATGGTATGTGTTGCC TT-3′). PCR cycling conditions were 10 min at 95°C for 1 cycle followed by 45 cycles at 95°C for 30 sec, 60°C for 30 sec and 72°C for 60 sec. Dissociation curve analyses confirmed that signals corresponded to unique amplicons. Expression levels were normalized to the glyceraldehyde-3-phosphate dehydrogenase (GAPDH) mRNA level of each sample, obtained from parallel assays.

## Results

### CD82 does not influence Wnt protein expression

**Figure 1:**
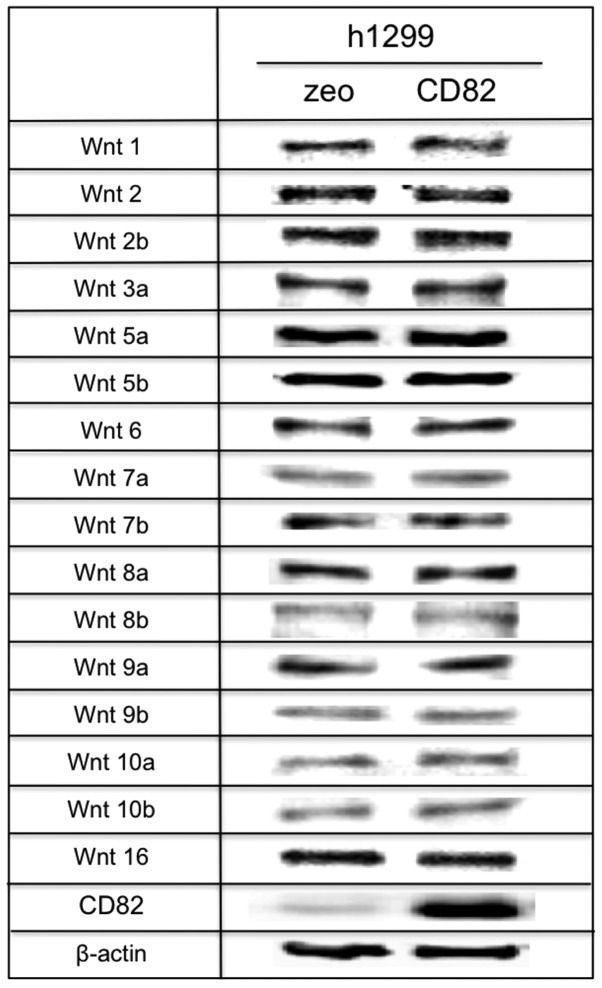
Effect of CD82 on the expression of Wnt proteins. Whole cell lysates (200 *μ*g) of h1299 cells [h1299/zeo (zeo), h1299/CD82 (CD82) h1299/CD82-sh. control (sh.cont) and h1299/CD82-sh.CD82 (sh.CD82)] were resolved by 10% SDS-PAGE and analysed by immunoblotting with the anti-Wnt antibodies indicated in the figure. The same blots were re-blotted with an anti-CD82 antibody and an anti-β-actin as loading control. Experiments were performed in triplicate and the most representative data are shown.

The cellular distribution of β-catenin is regulated by the Wnt/β-catenin (canonical) signalling pathway. This pathway is initiated by binding of Wnt ligands to their Fzd receptor proteins. To evaluate the effect of CD82 on Wnt signalling, we first analysed the protein expression levels of Wnt ligands on h1299 cells by immunoblotting. As shown in Fig. 1, h1299 cells expressed all classes of Wnt ligands (1, 2, 2b, 3a, 5a, 5b, 6, 7a, 7b, 8a, 8b, 9a, 9b, 10a and 10b). The expression of Wnt ligands was not significantly altered after ectopic expression of CD82 at the protein level.

### CD82 inhibits the expression of specific Fzd proteins

Next, we examined the expression of Fzd proteins, which are the transmembrane receptors for Wnt ligands (34). Binding of Wnt proteins to their Fzd receptors transduces Wnt signalling via inactivation of GSK-3β and consequent nuclear translocation of unphosphorylated β-catenin.

**Figure 2:**
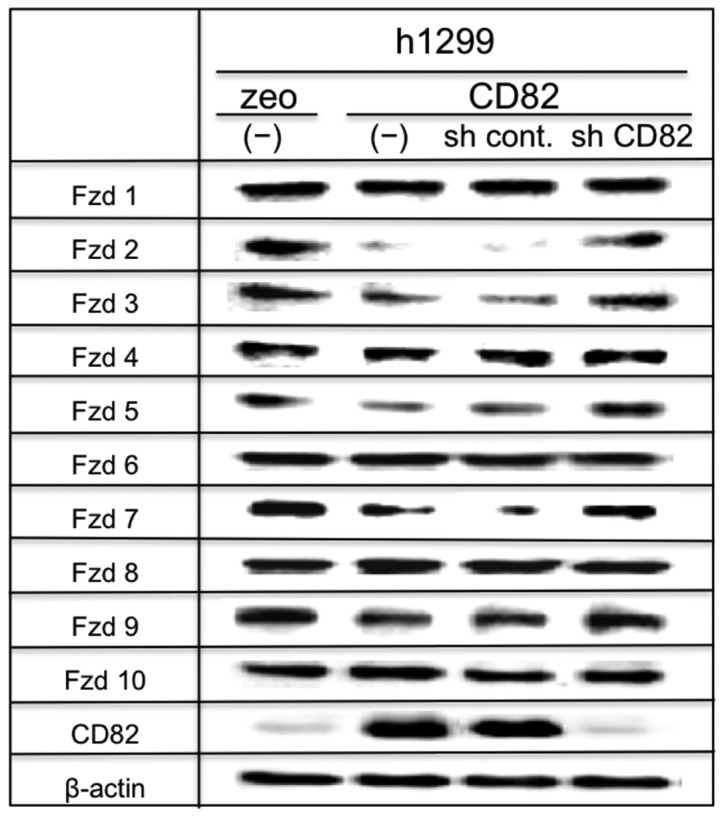
Effect of CD82 on the expression of Frizzled (Fzd) proteins. Whole cell lysates (200 *μ*g) of h1299 cells [h1299/zeo (zeo), h1299/CD82 (CD82), h1299/CD82-sh.control (sh.cont), h1299/CD82-sh.CD82 (sh.CD82)] were resolved by 7.5% SDS-PAGE and analysed by immunoblotting with the anti-Fzd antibodies indicated in the figure. The same blots were re-blotted with an anti-CD82 antibody and an anti-β-actin as loading control. Experiments were performed in triplicate and the most representative data are shown.

Fzd1-Fzd10 were expressed on h1299 cells. In our model, the expression of Fzd1, Fzd4, Fzd6, Fzd8 and Fzd10 was not markedly affected by CD82. In contrast, CD82 significantly downregulated the expressions of Fzd2, Fzd3, Fzd5, Fzd7 and Fzd9. Knocking down of CD82 mRNA expression by shRNA in h1299/CD82 cells resulted in recovery of the expression of those downregulated Fzd proteins, suggesting a specific effect of CD82 on Fzd2, Fzd3, Fzd5, Fzd7 and Fzd9 expression (Fig. 2).

This result suggests that CD82 reduces receptor binding of Wnt ligands by inhibiting Fzd expression, thereby reducing Wnt signalling and β-catenin translocation to the nucleus.

### CD82 controls β-catenin cellular distribution and inhibits β-catenin phosphorylation

In the absence of Wnt ligands or in the event of impaired Wnt signalling, cytosolic β-catenin is phosphorylated at Ser45 by CK1α. Consequently, GSK-3β phosphorylates β-catenin at Thr41, Ser37 and Ser33 (20). Ser33/Ser37 double-phosphorylated β-catenin is specifically recognized by β-TrCP (35) and rapidly degraded.

Therefore, we next examined the effect of CD82 on the cellular distribution and phosphorylation of β-catenin. We performed subcellular fractionation and determined the amount of total and phosphorylated β-catenin in h1299 cells by immunoblotting.

**Figure 3:**
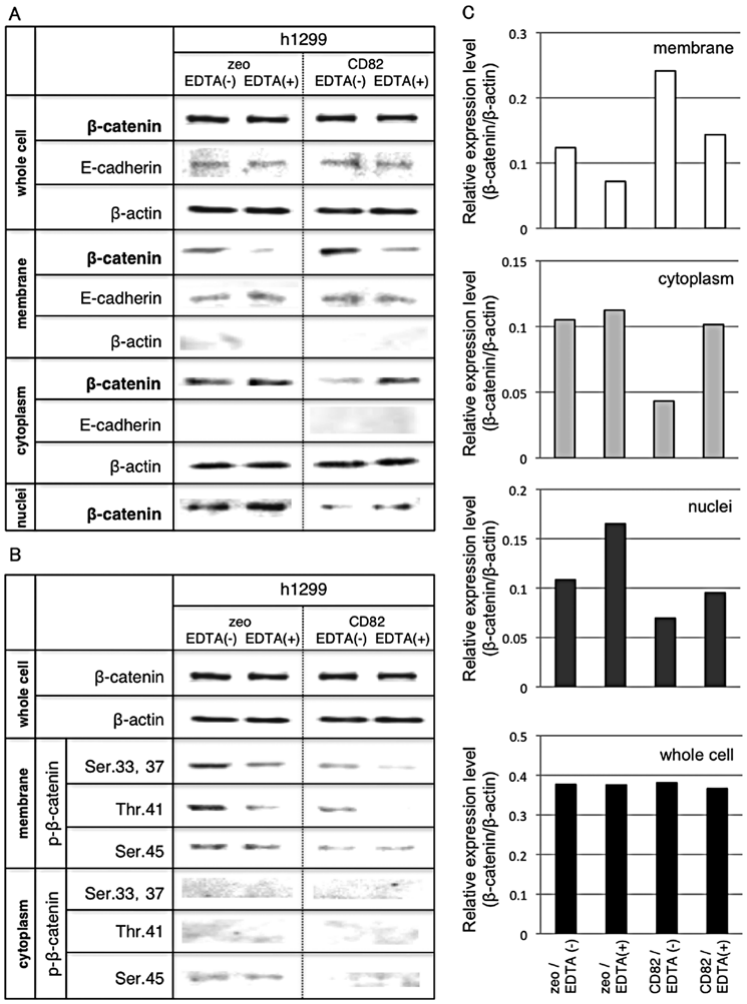
Effect of CD82 on β-catenin cellular distribution and phosphorylation. h1299 cells (4.5×10^6^) were cultured in serum-free medium containing 5% BSA (EDTA^−^) or in Ca^2+^- and serum-free medium containing 5% BSA with 0.5 mM EDTA (EDTA^+^) for 12 h, followed by subcellular fractionation. Extracted cellular fractions of h1299 cells were resolved by SDS-PAGE and analysed by immunoblotting with anti-β-catenin (A) and anti-phospho-β-catenin (Ser33, Ser37, Thr41 and Ser45) (B) antibodies. A densitometric analysis was performed on (A), followed by normalization to the densitometric value of β-actin and indicated as ‘relative expression value (β-catenin/β-actin)’ (C). Experiments were performed in triplicate and the most representative data are shown.

Fig. 3A shows the cellular distribution of β-catenin in h1299 cells. The quantitated data are shown in Fig. 3C. We found significant accumulation of β-catenin at the cell membrane of h1299/CD82 cells. From our previous studies that showed increase of the E-cadherin-β-catenin complex in h1299/CD82 cells (33), we used EDTA in serum-free medium in order to destabilize E-cadherin. Low Ca^2+^ treatment enhances E-cadherin internalization (which is independent of tyrosine phosphorylation and ubiquitination) and E-cadherin is recycled back to the plasma membrane (36). EDTA treatment reduced β-catenin at the cell membrane in both h1299/zeo and h1299/CD82 cells. The amount of cytosolic β-catenin in h1299/zeo cells was only mildly increased by EDTA treatment, whereas that in h1299/CD82 cells was remarkably increased. Nuclear β-catenin was basically decreased in h1299/CD82 cells. Interestingly, EDTA treatment significantly increased β-catenin levels in h1299/zeo cells but not significantly so in h1299/CD82 cells (Fig. 3A and C). These results support our previously reported results (33) and suggest that CD82 controls the distribution of cytoplasmic β-catenin to the membrane rather than into the nucleus.

Fig. 3B shows the levels of phosphorylated β-catenin in h1299 cells. The amount of phosphorylated β-catenin (Ser33, Ser37, Thr41 and Ser45) at the cell membrane was reduced by overexpression of CD82. In addition, EDTA treatment inhibited β-catenin phosphorylation (at Ser33, Ser37, Thr41 and Ser45) in both cell lines.

These data suggest that CD82 inhibits the cytoplasmic translocation and phosphorylation of β-catenin (Ser33, Ser37, Thr41 and Ser45) at the cell membrane. Moreover, CD82 reduces nuclear translocation of β-catenin even when E-cadherin is destabilized.

### CD82 inhibits GSK-3β and CK1α expression

Inhibition of β-catenin phosphorylation (Ser33, Ser37, Thr41 and Ser45) by CD82 also suggests that β-catenin may be differently phosphorylated by GSK-3β and CK1α.

**Figure 4:**
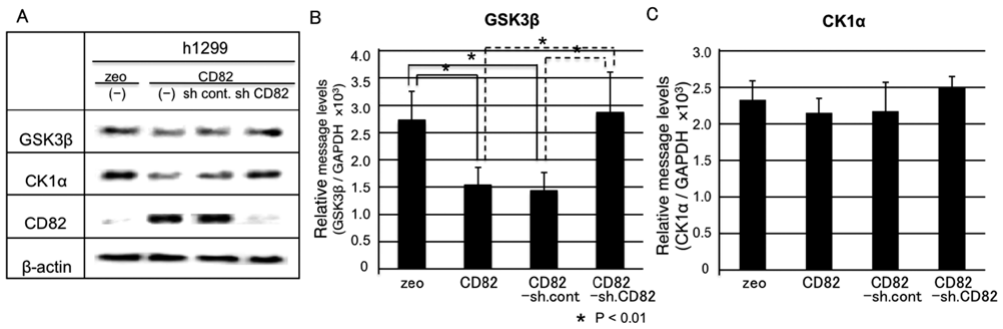
Effect of CD82 on the expression of GSK-3β and CK1α protein and mRNA. (A) Whole cell lysates (200 *μ*g) of h1299 cells [h1299/zeo (zeo), h1299/CD82 (CD82), h1299/CD82-sh.control (sh.cont), h1299/CD82-sh.CD82 (sh.CD82)] were resolved by 7.5% SDS-PAGE and analysed by immunoblotting with anti-GSK-3β and anti-CK1α antibodies. The same blots were re-blotted with an anti-CD82 antibody and an anti-β-actin as loading control. Experiments were performed in triplicate and the most representative data are shown. (B) Total RNA was isolated from h1299 cells and analysed by real-time RT-PCR. mRNA levels of GSK-3β were corrected relative to GAPDH mRNA. The asterisks in the figure indicate statistically significant difference (p<0.01) between the 2 values. Data are presented as the means ± SEM. (C) Total RNA was isolated from h1299 cells and analysed by real-time RT-PCR. mRNA levels of CK1α were corrected relative to GAPDH mRNA. Data are presented as the means ± SEM.

In a first step, we examined the protein expression levels of GSK-3β and CK1α by immunoblotting. The protein levels of GSK-3β and CK1α were downregulated by CD82 and this effect was reverted by knocking down CD82. These results indicate a specific effect of CD82 on GSK-3β and CK1α (Fig. 4A).

Next, we examined the mRNA levels of GSK-3β and CK1α by real-time RT-PCR. GSK-3β mRNA levels were significantly downregulated by CD82 and this downregulation reverted by knocking down CD82 (Fig. 4B). In contrast, CK1α mRNA levels were not significantly downregulated by CD82, but the slight downregulation was still reverted after knocking down CD82 (Fig. 4C). This result suggests that CK1α protein levels are inhibited by CD82 post-transcriptionally; however, the underlying mechanisms remain unknown.

## Discussion

In our previous research, we showed that CD82 inhibits tyrosine phosphorylation of β-catenin and stabilizes E-cadherin-β-catenin complexes at the cell membrane (33). CD82 induces homocellular adhesion and controls cellular distribution of β-catenin to the cell membrane rather than to the nucleus. It has been shown that cellular β-catenin levels are regulated at 3 different levels: the first is regulation by RTK (37,38); the second, regulation by the Wnt/β-catenin (canonical) pathway (18); and the last, control by endosomes and exosomes (39–41). We have also shown that CD82 inhibits β-catenin phosphorylation via the epidermal growth factor receptor (EGFR) and c-Met (33). This is a novel function of CD82 in E-cadherin-mediated homocellular adhesion and one of the most important functions of CD82 in inhibiting cancer metastasis. In this study, we further found evidence that CD82 regulates β-catenin cell distribution by inhibiting Wnt signalling at multiple molecular levels.

All members of the Wnt protein family are extracellular secreted proteins. Binding of a Wnt ligand to its co-receptors Fzd and LRP5/6 results in the activation of Dvl, which in turn inhibits GSK-3β-mediated phosphorylation of β-catenin (19). We could not find any differences in the protein expression of Wnt proteins after ectopic expression of CD82. On the other hand, we found significant downregulation of Fzd2, Fzd3, Fzd5, Fzd7 and Fzd9 after CD82 ectopic expression. This effect was reverted by shRNA knock-down of CD82, suggesting the specific effect of CD82. Although the specificity of the Wnt signalling is determined by the interaction of specific pairs of Wnt and Fzd proteins, the mechanisms remain to be elucidated. However, in various types of cancer cells, it has been reported that particular Wnt-Fzd interactions are important in tumour progression (18). In particular, binding of Wnt5a to Fzd2 or Fzd7 controls metalloproteinase production (42,43) and focal adhesion dynamics via the Wnt signalling pathway (44). It has also been reported that Wnt5a expression is of clinical relevance in prostate cancer (42). These reports support the idea that Fzd2, Fzd3, Fzd5, Fzd7 and Fzd9 (down-regulated by overexpression of CD82) are key players in many types of cancer cells. Therefore, specific downregulation of Fzd receptors by CD82 may reduce Wnt/β-catenin signalling. Furthermore, Wnt target genes (Wnt3a, Fzd7, Axin, TCF/LEF, among others) are all molecules related to Wnt signalling itself (45–49). Inhibition of Wnt signalling pathway leads to further negative regulation of the pathway. This results in inhibition of β-catenin nuclear translocation and consequent accumulation in the cytoplasm.

Accumulated cytoplasmic β-catenin is phosphorylated at Ser45 by CK1α and incorporated into a cytosolic protein complex containing Axin, APC and GSK-3β. GSK-3β phosphorylates β-catenin at residues Ser33, Ser37 and Thr41 (20), thereby targeting it for ubiquitination by β-TrCP and subsequent degradation in the proteasome. Ectopic expression of CD82 inhibits phosphorylation of β-catenin at Ser45, Ser33, Ser37 and Thr41 by downregulation of GSK-3β and CK1α, thereby favouring the escape of β-catenin from the ubiquitination and degradation process. Therefore, downregulation of GSK-3β and CK1α by CD82 ultimately leads to accumulation of β-catenin in the cytoplasm. Contradictorily, we found a decrease in cytoplasmic β-catenin and a significant increase in β-catenin accumulation at the cell membrane of CD82-transfected cells (Fig. 3A). We also found that EDTA-destabilized E-cadherin inhibits the translocation of β-catenin to the cell membrane (Fig. 3A). This result suggests that β-catenin, when at the cell membrane, is associated with E-cadherin. We have already shown that overexpression of CD82 induces interaction between E-cadherin and β-catenin and one possible mechanism is the inhibition of RTK signalling pathway by CD82. CD82 inhibits EGFR tyrosine phosphorylation (32) and Ras- and PI3K-dependent c-Met signalling (50). Direct association of CD82 with these growth factor receptors inhibits receptor signal transduction, which will result in inhibition of β-catenin tyrosine phosphorylation (50). Several studies have shown that either increased kinase activity through stimulation with EGF or decreased phosphatase activity using pervanadate or phosphatase mutants leads to decreased interaction between cadherin-catenin complexes and the cytoskeleton. CK1α also destabilizes E-cadherin by phosphorylation of the cytoplasmic domain of E-cadherin. Downregulation of CK1α by CD82 overexpression will result in E-cadherin stabilization (51). Therefore, CD82 strengthens the interaction between E-cadherin and β-catenin by multiple pathways and results in translocation of accumulated cytoplasmic β-catenin to the membrane.

Furthermore, this result also serves as evidence to show that CD82 inhibits the nuclear translocation of β-catenin. Accumulation of β-catenin in the cytoplasm will result in nuclear translocation of β-catenin, as observed in the EDTA-treated h1299/zeo cells (Fig. 3A). In contrast, EDTA treatment actually increased β-catenin in the cytoplasm while impairing its translocation to the nucleus. These findings highlight an important function of CD82 in inhibiting β-catenin translocation to the nucleus. However, the mechanism remains to be elucidated.

Recently, a novel inhibitory mechanism of Wnt signalling by CD82 was described. CD82 and other tetraspanins are organized in a signalling complex with E-cadherin at the plasma membrane. This signalling complex, including tetraspanins, E-cadherin and β-catenin, is internalized and delivered to early endosomes (41). Exosome biogenesis begins with outward vesicle budding at the limiting membrane of endosomes, generating intraluminal vesicles (ILVs). These exosome-containing endosomes eventually mature into late endosomes, also known as multivesicular bodies (MVBs). These MVBs then fuse with the plasma membrane and release their intraluminal vesicles, referred to as exosomes, which contain β-catenin. Exosomal targeting of β-catenin causes a reduction in the intracellular pool of β-catenin and therefore reduces Wnt/β-catenin signalling. This mechanism is thought to occur after CD82-induced β-catenin membrane translocation, as shown in this study. Therefore, CD82 recruits β-catenin and E-cadherin to the plasma membrane, thereby contributing to the formation of large signalling complexes, which are in turn incorporated into β-catenin-containing exosomes, whose contents are released outside the cells.

**Figure 5:**
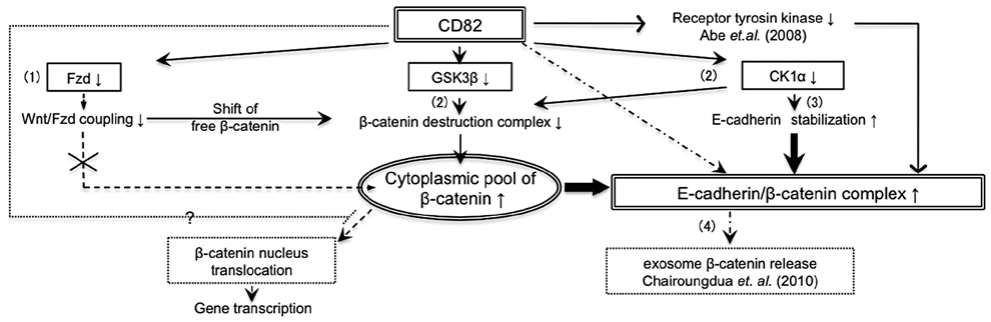
CD82 attenuates Wnt signalling at multiple levels (working hypothesis). CD82 attenuates Wnt signalling in multiple ways: i) inhibition of β-catenin nuclear translocation by downregulation of Fzd receptors or other mechanism; ii) accumulation of β-catenin in the cytoplasm is accelerated by downregulation of GSK-3β and CK1α; iii) recruitment of β-catenin to the cell membrane by stabilization of the E-cadherin-β-catenin complex by inhibition of RTK and downregulation of CK1α; and iv) CD82 induces exosomal β-catenin release as described by Chairoungdua *et al*(41).

Altogether, CD82 attenuates Wnt signalling in multiple ways (Fig. 5): i) inhibition of β-catenin nuclear translocation by downregulation of Fzd receptors or other mechanism; ii) accumulation of β-catenin at the cell membrane by downregulation of GSK-3β and CK1α; iii) stabilization of the E-cadherin-β-catenin complex by inhibition of RTK and downregulation of CK1α; and iv) induction of exosomal release of β-catenin. In the first step, (i) and (ii) will increase the cytoplasmic pool of β-catenin, whereas in the second step, (iii) and (iv) will reduce the cytoplasmic pool of β-catenin. Inhibition of Wnt signalling by β-catenin translocation but not by β-catenin degradation leads to E-cadherin stabilization at the cell surface and strengthens homophilic adhesions between cancer cells.

In conlusion, CD82 suppresses cancer metastasis through the canonical Wnt pathway via multifunctional ways. Down-regulation of Fzd receptors also suggests inhibition of the non-canonical Wnt pathway. However, this mechanism remains to be elucidated. Overall, accumulating evidence of the anti-metastatic effect of CD82 suggests CD82 as a novel therapeutic target for anti-metastasis cancer therapy.
